# Behavior of Children during Dental Care with Rubber Dam Isolation: A Randomized Controlled Study

**DOI:** 10.3390/dj9080089

**Published:** 2021-08-04

**Authors:** Tania Vanhée, Chloé Tassignon, Pierre Porta, Peter Bottenberg, Thierry Charles, Astrid Vanden Abbeele

**Affiliations:** 1 Department of Paediatric Dentistry, Université Libre de Bruxelles, 1070 Brussels, Belgium; chloe.tassignon@gmail.com (C.T.); porta.pierre@gmail.com (P.P.); Astrid.Vanden.Abbeele@ulb.be (A.V.A.); 2 Oral Health Research Group, Faculty of Medecine and Pharmacy, Vrije Universiteit Brussel, 1070 Brussels, Belgium; pbottenb@vub.be; 3 Department of Endodontics, Université Libre de Bruxelles, 1070 Brussels, Belgium; charles.th@gmail.com

**Keywords:** behavior, rubber dam, pediatric dentistry

## Abstract

Background: The establishment of the dental dam improves dentist working conditions and patient protection. The purpose of this study was to analyze the behavior of the child during dental care with or without a dam. Methods: In this interventional randomized study, 51 patients are divided into two groups, one with a rubber dam and the other with cotton roll isolation. Their behavior was observed during the treatment of temporary molars. The duration of the treatment, the patient’s feeling with a visual analogue scale (VAS), the behavior (B) of the child measured with a hetero-evaluation scale (modified Venham scale) and the cardiac frequency (CF) were measured. Results: The group treated with a rubber dam has a significant decrease in the various stress parameters that have been identified (B, *p* value = 0.034; CF, *p* value = 0.015). Subgroups of patients with and without nitrous oxide sedation were compared and similar results were obtained. Conclusions: Isolation with a rubber dam reduces child’s stress during dental care. Although it is slightly more time-consuming and training is necessary for a quick and effective placement, it allows dentists to perform dental care in the best possible conditions, while reducing dental anxiety in young patients.

## 1. Introduction

The dental dam, designed in 1864 by Dr. Stanford C. Barnum, is a square of latex that isolates one or more teeth from the rest of the oral cavity to work away from saliva. Hence, dental care could be rendered in better working conditions: absence of saliva, better visibility, and asepsis [1]. The rubber dam (RD) also protects the patient against ingestion or inhalation of potentially toxic mucosal and aerosol products containing pathogens [2]. This aspect is even more important in the context of the COVID-19 pandemic: rubber dam reduces by 70% the production of droplets or aerosols contaminated by the patient’s saliva or blood within 1 m radius [3].

While the use of the RD was often associated with endodontic treatments and adhesive procedures, RD has been used as an essential aid during treatment of pediatric dental patients several decades ago [4,5]. However, previous studies in the literature do insufficiently highlight the benefits of good insolation on the quality of bonding [6,7].

The majority of non-food-type foreign body ingestions occurs in children [8]. Uncooperative children or mentally or physically handicapped children were more likely to swallow or aspirate instruments during dental care [9].

The positioning of a dental dam not only creates better working conditions but also ensures the protection of the patient. If it is interesting to use the dam for all the reasons mentioned above, how does the child feel about the care administered under the dam? Does the presence of the dam increase the stress of the child or reduce it? McKay published a study on the feeling of the patient after the treatment. The results showed that, for the most part, the dam is well accepted by children [10]. In addition, several studies have shown that adult patients prefer dental treatment with a rubber dam than without [11,12].

If acceptance is one outcome variable, levels of anxiety have been studied in another study and shown no difference between treatment with or without rubber dam [13]. However, stress indicators in children were not yet reported. This raises the question: can the establishment of the dam reduce or induce stress during dental care in children?

This study aims to determine the behavioral and physiological indicators of stress in children during dental care with or without a rubber dam with the hypothesis that rubber dam decreases stress. The null hypothesis is that there is no difference between the outcomes measured in the two groups.

## 2. Materials and Methods

Based on the sample size of comparable studies, 51 children from 3 to 10 years old were recruited from a pool of patients consulting in the pediatric dentistry department of CHU Saint-Pierre, César de Paepe site (Brussels, Belgium), during the period from 17 November 2017 to 07 March 2018. Patients needing operative treatment on at least one primary molar were eligible to participate. Children with infected or mobile decayed teeth as well as those outside the age range studied were excluded.

The procedures followed were in accordance with the ethical standards of the responsible committee on human experimentation (institutional and national) and with the Helsinki Declaration of 1975, as revised in 2000. All patients and their legal guardians who agreed to participate in the study signed an informed consent form. The study was validated by the ethics committee of CHU Saint-Pierre: B076201734515.

The patients included in the study were randomized at the beginning of the day by random draw and assigned either the test group with rubber dam (RD) or the control group with cotton roll isolation (CR). Treatment was performed by 12 practitioners belonging to the pediatric dentistry team. All operators have been trained during their studies on the RD placement and are accustomed to the procedure. Among the 12 operators, there were 2 seniors (18 patients), 5 post-graduates (16 patients) and 5 master’s students (17 patients).

When judged necessary by the operator, treatments were performed under conscious sedation (CS) using a premixed oxygen/nitrous oxide gas.

All treatments were followed by a single observer who collected the data (TC) and was previously trained in behavioral evaluation by the modified Venham hetero-evaluation scale (VS). The training was carried out prior to the experimentation by estimating behavior on 10 videos of children with different attitudes towards the dental care received. The VS describes the different stages of the child’s behavior during dental care, from the fully relaxed child (score 0), to the concerned child but able to cooperate (score 1), the tensed child with the undisturbed continuity of the treatment (score 2), the reluctant child who has a dental treatment with difficulties (score 3), the very disturbed child who regularly protests and disrupts the procedure (score 4) and the completely disconnected and untreatable child (score 5) [14].

In the RD group, patient’s cardiac frequency (CF) was recorded at the same time points using a Digital Finger Pulse Oxygen Saturation Monitor OLED display (Elera, China) [15].

In the RD group, data were recorded at five different time points (T0 to T4). T0: patient installed in the dental chair (in CS group after 3 min of inhalation), T1: during local anesthesia, T2: before placing the rubber dam, T3: with the dam installed and T4: during the treatment.

In the CR group, the same parameters were recorded at T0, T1 and T4 only, since no rubber dam was used.

At the end of the treatment session, total duration from T0 to T4 was recorded as well as the feeling of the patient after the intervention measured with a visual analogue scale (VAS), graduated from 0 to 10, 0 being the absence of pain or stress.

The results were transferred to a computer for further data analysis. Data sets from all participants were complete. Descriptive data were established. Further statistical tests were performed using SPSS (IBM, Armonk, NY, USA) and Prism 6 (GraphPad, LaJolla, CA, USA) software. Significance was accepted at a *p*-value equal or lower than 0.05. Data were compared within groups using paired tests and between groups using unpaired tests. Furthermore, differences between time points were compared to 0 by a one-sample test. Categorical data (Venham scale, VAS) were analyzed using non-parametric tests.

Trial registration: Retrospectively registered 39,217 in ISRCTN Register (London, UK) with ISRCTN15046229. The first registration was made on 7 January 2021.

## 3. Results

A total of 51 children between 3 and 10 years old, 21 girls and 30 boys, were randomly divided into two groups: 24 patients for the RD group and 27 patients for the CR group. The average age of both groups was 6.55 years.

A total of 30 patients were treated without conscious sedation (CS) and 21 with CS, of which 9 with RD and 12 with CR (Chi2, *p* = 0.78). The behavior (VS) and cardiac frequency (CF) at T0 did not show a significant difference between the 2 groups (CF: Student’s *t*-test, VS: Mann–Whitney test) (Table 1).

The average treatment duration was 15 min 47 s for the CR group and 17 min and 54 s for the RD group (*p* = 0.02, Student’s *t* test). The patients evaluated their treatment experience via the VAS as generally positive (RD: median 0, IQR 0–1; CR 0, IQR 0–5). There is no significant difference between the two groups.

**Figure 1:**
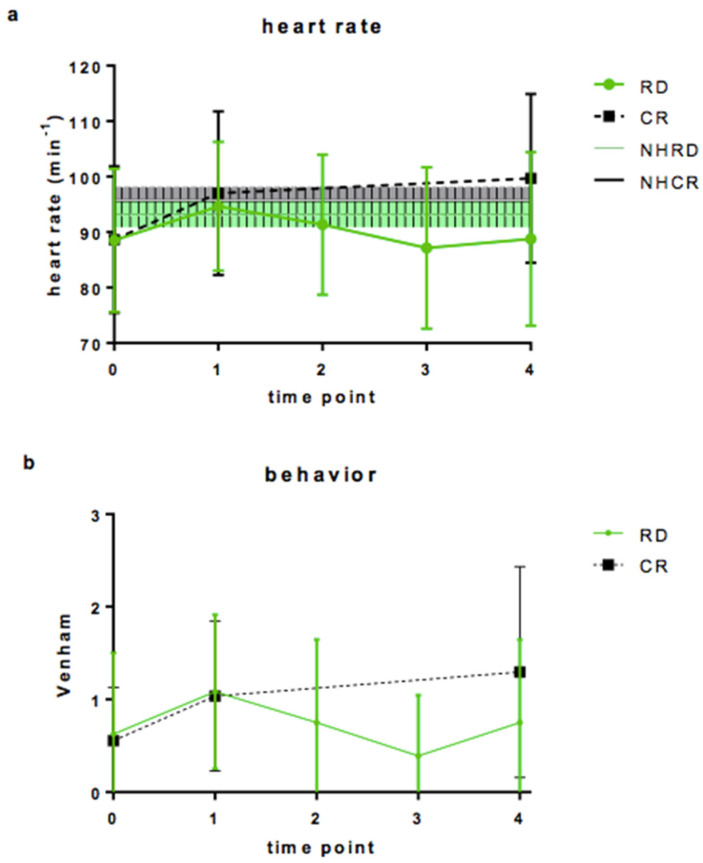
(**a**): Heart rate (min^−1^) during the treatment with rubber dam (RD) or cotton-roll (CR) isolation. The shaded area represents the median and interquartile range of mean heart rate of the children (according to Fleming et al., 2011), (green RD, grey CR) at the different time points: T0: before intervention, T1 after local anesthesia, T2: before rubber dam placement, T3: after rubber dam placement, T4 during procedure. (**b**): Venham scale recorded at the same time points.

In Figure 1, the outcomes at different time points are shown. In the CR group, CF and Venham scale tended to increase from T0 to T4, while in the RD group this was not the case.

The heart rate is not significantly different from the median resting values per age group [15] during the treatment in both groups. Venham scale values increased in both groups while anesthetic administration and even more during intervention in the CR group. In the RD group, Venham scale values decreased after T1 and stayed around initial level further on.

Differences in vs. and HR between different time points are shown in Table 2. Heart rate and vs. increased significantly between T0 and T4 in the CR group and did not in the RD group (Table 2, part 1). There is a significant moderate Spearman rank correlation between heart rate and vs. at T4 with a correlation coefficient of 0.458 (*p* < 0.01), but not significant at the other time points.

After rubber dam placing, heart rate and vs. dropped significantly (Table 2, part 2, CF: Student’s *t*-test, vs.: Mann–Whitney test; *p* < 0.05).

Among the patients observed (n = 51), 21 of them were treated with conscious sedation (CS). The evolution of anxiety indicators by the T4-T0 comparison is analyzed in subgroups with CS and without CS.

In the RD group, heart rate did not increase compared to T0: it even decreased slightly in the CS/RD group.

Contrarily, heart rate increased significantly in the CR group, whether CS was administered or not. vs. difference was significantly lower in the RD/CS group compared to CR/CS. (CF: ANOVA test, vs.: Kruskal–Wallis test) (Table 2, Part 3). Rubber dam combined with conscious sedation showed the lowest anxiety indicators.

## 4. Discussion

One aim in pediatric dentistry is to allow our young patients to better experience dental care with comfort so that a relation of confidence is created with their pediatric and later general dental practitioner. Teaching of the RD practice in (under-)graduate training was primarily intended for technical reasons and asepsis of the dental operating field, particularly in endodontics. However, the clinical experience with this tool has shown an interesting side effect: relaxation and, if the treatment lasts a long time, slumber. This empirical observation has been made for both adults and children.

Over the years, the use of the dam has no longer been exclusively indicated for endodontics or adhesive procedures. As students and practitioners acquire more skills in rubber dam use in adults, the step towards its application in the field of pediatric dentistry can easily be made. Our results show that the extra time needed for placing, adapting and removing rubber dam was about 2 min. As result of this small extra time, patient and practitioner gain in ergonomics: no more discomfort at the level of cotton rolls and the saliva ejector which reduce space in the operating field, lips, cheek and tongue are spread flexibly unlike manual retraction, no more saliva contaminating the treated tooth. However, on top of that, the patient relaxes and sometimes falls asleep. During this research work, the objective was to shed light on this clinical observation by scientific evidence. Observation of behavior and heart rate of patients with or without a dam was chosen in order to have a simple, reproducible and clinically sensible protocol.

Whether in terms of behavior or heart rate, a favorable outcome in the RD group was observed compared to the CR group. This tendency is even more marked in patients observed in conscious sedation. Indeed, if the values recorded at different times in patients treated in the vigil state decrease after the stress peak of local anesthesia (T3), this decrease is even more important in patients in conscious sedation to the point that the patient is more relaxed than entering the dental office. Such a decrease is not observed with CR. The RD brings a state of relaxation to the patient, observable at the behavioral level and the heart rate. These same trends have been observed in other studies [16,17].

One of the most comparable studies with ours is that of Ammann et al. Our results point in the same direction, even if two fundamental elements are very different—the operator and the act itself. Ammann et al. mention the possibility of bias linked to the practitioner’s preference for providing care under a dam [18]. A total of 12 different practitioners participated in our study; this diversity excludes the bias of the single practitioner. Moreover, in this study, only fissure sealants were placed, a preventive act that did not require local anesthesia. However, we know that placing the RD clamp can be painful and, therefore, represents an observation bias in terms of dental anxiety.

Indeed, an RD disadvantage is that it requires local anesthesia (LA) because the placement of the clamp causes significant discomfort that is not present when using CR [17]. In the context of our study, all procedures were performed under LA. In other studies, the effect of the RD has been observed with pits and fissures sealings, an act that does not require local anesthesia. This choice of procedure is a bias in terms of the effect of the RD on anxiety because the placement of the clamp without LA is annoying or even painful. For technical procedures that do not require LA, a recent study has shown the interest of applying topical anesthetics to improve comfort when applying the clamp [19]. Other systems have also been studied, such as Isolite^®^ [20].

For children, local anesthesia is a source of anxiety. During anesthesia, the score obtained on the Venham scale rises by one or two graduations, even in the most relaxed children. However, the interest of this study was to assess the effect of the dam on the anxiety of the young patient. The choice of a treatment with local anesthesia due to the nature of the treatment then made ethical sense.

During the treatment, if noises generated by rotary instruments are the same in both procedures, the suction cannula inconveniences are considerably decreased as soon as the dam is put in place. There was a decrease in the Venham scale or a return to the base level (T0). In the CR group, a persistent stimulus such as water flowing in the mouth or the taste of certain products may contribute to a further rise of stress-related behavior [21].

In our study, anxiety indicators were reduced by the use of the rubber dam during the dental procedures when compared to cotton-roll insolation.

How to explain this reduction of stress in children? “Anxiety is a reaction induced by our primary brain. It decreases by using the dam because it helps to reduce or even eliminate the feeling of rape of his intimacy. The patient no longer feels the intrusion, the tooth is like outside of the mouth” [6].

The dam provides a feeling of protection, as if the care was happening outside the mouth [18].

The relaxation of the patient illustrated by the results obtained corresponds to what can be observed during clinical practice. The patient has a feeling of dental care being much less invasive, there is less effort to keep the mouth open during the treatment, and less inconvenience by water or by suction in contact with tongue or lingual floor. If the treatment takes a little longer, some patients even tend to fall asleep. No case of falling asleep was observed in our study because the operative criterion was to be limited to conservative treatments on deciduous molar which were carried out over a fairly short period, less than 20 min on average.

The behavior of the child at different times was evaluated in this study. Most children (46/51) were between 0 and 1 throughout the treatment. The difference between the two levels (0 and 1) was hardly noticeable because the stress of the child can only materialize by a tight hand, a worried look, moving feet or other signals. Since these differences in behavior were difficult to discern, the use of heart rate measurement was very relevant. Even if the subgroups of samples were small in terms of the analysis of patients treated under conscious sedation, our study showed a very clear effectiveness of the rubber dam combined with conscious sedation. We could demonstrate a correlation between heart rate and VS, although it was not perfect. Other possible stress indicators were cortisol in saliva [22]. However, heart rate can easily be measured, especially in patients undergoing conscious sedation.

Cardiac frequency (CF) is considered as a very representative parameter of stress. The autonomic nervous system is broken down into two nervous systems, the sympathetic nervous system (exciter) SNS and the parasympathetic nervous system (inhibitor) PNS. In his study, Appelhans showed that in a state of physical or psychological stress, the activity of the SNS becomes dominant. It then produces an excitation and, therefore, an increase in CF. PNS activity dominates during periods of rest or safety and then causes a decrease in CF. [21] Therefore, a physical or psychological stress causes the activation of the excitatory nervous system which increases the CF. In our study, there is a highly significant difference (*p* = 0.001) in CF that decreases when a dam is used during care [23].

If this system brings so much benefit, one might wonder why practitioners tend to use it so little. Indeed, the RD currently remains an underused system [24]. The majority of clinical situations where the dental dam is used indicate the value of the mechanical barrier to avoid salivary contamination during bonding or endodontic treatment [25]. In our study, the use of RD has been analyzed as a tool for managing dental anxiety, a tool accessible to any practitioner. If the dental dam requires training for easy installation, master’s students are quite capable of placing it even despite their experience in very short dental practice [26]. The argument of loss of time during treatment is also often put forward [2]. However, in our study and, whatever the level of expertise of the practitioner, the average duration of treatment is greater than 2 min for the RD group, which is explained by the implementation of an additional procedure. The cost of the material is not an argument in Belgium because the nomenclature in our country provides for appropriate pricing and reimbursement.

## 5. Conclusions

The results obtained in this study show that the use of the rubber dam allows to reduce the stress in young patients during dental cares.

## Figures and Tables

**Table 1:** Overview of the cohort properties, per total of patients or different subgroups at start of the experiment. Difference between CR and RD groups were tested, and results are given with the applied test (X2: chi2, t: student’s *t*-test, MW: Mann–Whitney test).

Variable	CR Group	RD Group	Total/Mean	*p* Value
n	27	24	51	-
Girls (n)	8	13	21	X^2^, *p* = 0.08
Boys (n)	19	11	30	
Care with CS (n)	12	9	21	X^2^, *p* = 0.78
Care without CS (n)	15	15	30	
Mean age (years ± SD)	6.22 (1.81)	6.92 (1.73)	6.55 (1.81)	t, *p* = 0.18
Invasiveness (median, IQR)	3 (2: 5)	3 (3; 5.5)	3 (3: 5)	MW, *p* = 0.48
Behavior T0 (median, IQR)	0 (0; 1)	0 (0; 1)	0 (0; 1)	MW, *p* = 0.84
Heart rate T0 (min^−1^ ± SD)	88.67 (12.97)	88.54 (12.68)	88.6 (12.96)	t, *p* = 0.97

**Table 2:** Behavior according to Venham (VS) and Cardiac frequency (CF) comparisons for subgroups undergoing procedures with or without conscious sedation. *p*-value concerns CR and RD groups for part 1 and 3 and before and after rubber dam placement in RD group fort part 2 according to t Student test.

**Part 1: Before (T0) and during the Treatment (T4)**				
		CR group	RD group	
Mean difference (95% CI)		T4-T0	T4-T0	*p* value
VS		0.74 (0.35; 1.13)	0.13 (−0.31;0.56)	0.034
CF		11.04 (7.38; 14.69)	0.25 (−4.96; 5.46)	0.001
**Part 2: Before (T2) and after Rubber dam Placement (T3) in RD Group**				
Mean (SD)		Before Dam	After Dam	Statistics
VS		0.5 (0;1)	0 (0;1)	MW, *p* = 0.02
CF		91.33 (12.37)	87.13 (14.27)	t, *p* = 0.003
**Part 3: Before (T0) and during Treatment (T4) ** **with and without Nitrous Oxide Conscious Sedation (CS)**				
		CR group	RD group	
Mean (SD)		T4-T0	T4-T0	Statistics
VS	CS	1 (0; 1) *^, a^	0 (−1; 0) ^b^	KW, *p* < 0.05
	No CS	1 (0; 1) *^, a^	0 (0; 1) ^b^	
CF	CS	13.1 (8.6; 17.6) *^, a^	−0.9 (−10.3; 9.1) ^b^	ANOVA, *p* < 0.01
	No CS	9.4 (3.5; 15.3) *^, a^	0.9 (−5.9; 7.8) ^a^	

^a, b^: Superscript letters denote groups not significantly different from each other (*p* > 0.05) *: difference > 0, one-sample test, *p* < 0.05.

## Data Availability

Data available in a publicly accessible repository. The data presented in this study are openly available at this link: https://www.isrctn.com/ISRCTN15046229 accessed on 19 June 2021.
